# Endophthalmitis after tooth extraction in a patient with previous perforating eye injury

**DOI:** 10.11604/pamj.2015.20.72.6080

**Published:** 2015-01-27

**Authors:** Tevfik Ogurel, Zafer Onaran, Reyhan Ogurel, Kemal Örnek

**Affiliations:** 1 Kirikkale University Medical Faculty, Department of Ophthalmology, Kirikkale, Turkey

**Keywords:** Endophthalmitis, tooth extraction, perforating injury

## Abstract

The aim of this stuty is to describe a case of endophthalmitis after tooth extraction in a patient with previous perforating eye injury. 50 years old male patient attempted to our clinic with complaints of sudden severe pain, reduced vision, light sensitivity and redness in the right eye. The patient stated that severe pain in his eye began approximately 12 hours following tooth extraction. The patient's ocular examination revealed a visual acuity of hand motion in the right eye. Anterior segment examination of the right eye showed intense conjunctival hyperemia, chemosis, a fine keraticprespitat and corneal edema. Dental procedures of the patients who had recently underwent ocular surgery or trauma should be done in a more controlled manner under anti -infective therapy or should be postponed in elective procedures.

## Introduction

Endophthalmitis implies infection of the vitreous compartment together with the retinal and uveal coats of the eye. It may present as endogenous or exogenous infection, involving either following intraocular surgery (such as cataract, glaucoma, or occasionally squint) when pathogens harbored on the lids and conjunctival sac or following penetrating injury to the eye. Endogenous or metastatic endophthalmitis, a severe vision -threatening intraocular infection, occurs through bloodstream spread from a concurrent infection in the host or an external source. In this case report we present a patient developed endophthalmitis following tooth extraction who previously underwent primary repair surgery due to perforating eye injury two months ago.

## Patient and observation

**Figure 1:**
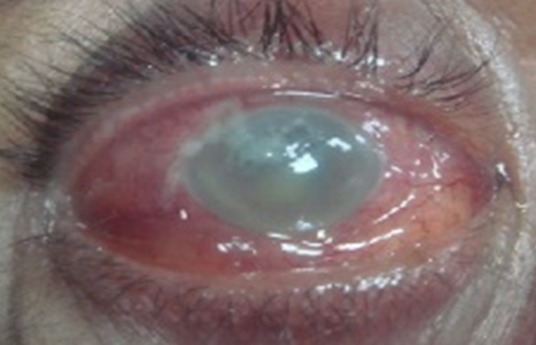
Right eye of the patient with endogenous endophthalmitis following pars plana vitrectomy showing intense conjunctival hyperemia, chemosis, corneal edema

50 years old male patient attempted to our clinic with complaints of sudden severe pain, reduced vision, light sensitivity and redness in the right eye. The patient underwent primary corneal suturation and one week later extra capsular cataract extraction because of perforating injury of the eye and traumatic cataract in our clinic two months ago. The patient stated that severe pain in his eye began approximately 12 hours following tooth extraction. The patient's ocular examination revealed a visual acuity of hand motion in the right eye. Anterior segment examination of the right eye showed intense conjunctival hyperemia, chemosis, a fine keraticprespitat, corneal edema and 10.0 corneal sutures in old corneal incision. Right eye was aphakic and illuminated fundus was blurred because of the corneal edema. Intense vitreous opacities were detected on ultrasound in the right eye. The left eye examination was unremarkable. Patient was diagnosed as endophthalmitis. He underwent vitreous tap and was injected 0,1 cc vancomycin/ 0,1 cc amikacin and topical fortified antibiotics (gentamycin -cefazolin) were initialized. The vitreous material was sent to microbiologic examination. Next day pars planavitrectomy was performed because of decreased vision to light perception on examination (Figure 1). Microbiological culture results were negative. In follow - up examinations he had no light perception and resulted in a phthisiceye.

## Discussion

Endogenous bacterial infection is relatively rare, accounting for 2% to 8% of all endophthalmitis cases [1]. In many patients accompanying systemic diseases such as diabetes, hypertension, gastro-intestinal disorders, cardiac disorders, malignancy and immunosuppression or else prolonged surgical complications are present with endophthalmitis [2]. Endogenous endophthalmitis can be developed from the focus of infection in any part of the body as well as also may develop from normal flora after surgery as a result of hematogenous spread. Our case is a phenomenon occurring after tooth extraction. As previously reported cases of endogenous endophthalmitis emerged after teeth cleaning and endogenous source of infection that could not found any infection focus in the body [3]. In contrast, focused on the normal flora elements of the oropharynx and nasopharynx may be caused to endogenous endophthalmitis [3]. In our case, focus of infection was not detected in the culture and examination of the patient and did not revealed any risk factors other than previous ocular surgery. Ocular complications after dental procedures are quite rarely reported in the literature. One of them is recurrent septic retinal embolism reported after infected tooth extraction [4]. Another case that was represented was endogenous endophthalmitis with sub-retinal abscess after treatment of gingival abscess [5]. As in our case and previously published cases, detailed history of the patient would accelerate the diagnosis of endophthalmitis which could be in endogenous form. Generally a bacteremia that occurs in a healthy person is unlikely to result in endophthalmitis. However, development of such a situation in our case can be explained by the hypothesis that a recent ocular trauma or surgery may provide a basis for the adherence of bacteria.

## Conclusion

Dental procedures of the patients who had recently underwent ocular surgery or trauma should be done in a more controlled manner under anti -infective therapy or should be postponed in elective procedures.

## References

[1] Zhang YQ, Wang WJ (2005). Treatment outcomes after pars plana vitrectomy for endogenous endophthalmitis. Retina..

[2] Lakosha H, Pavlin CJ, Lipton J (2000). Subretinal abscess due to Nocardia farcinica infection. Retina..

[3] Subramanian ML, Topping TM (2003). Endogenous endophthalmitis after routine dental cleaning. Arch Ophthalmol..

[4] Kilmartin DJ, Barry P (1996). Recurrent septic retinal emboli following dental surgery; Br Recurrent septic retinal emboli following dental surgery. Br J Ophthalmol..

[5] Tsai TH, Yang CH, Yang CM, Chen MS (2005). Endogenous endophthalmitis with subretinal abscess after dental procedures. J Formos Med Assoc..

